# Trk1-mediated potassium uptake contributes to cell-surface properties and virulence of *Candida glabrata*

**DOI:** 10.1038/s41598-019-43912-1

**Published:** 2019-05-17

**Authors:** Vicent Llopis-Torregrosa, Catarina Vaz, Lucia Monteoliva, Kicki Ryman, Ylva Engstrom, Attila Gacser, Concha Gil, Per O. Ljungdahl, Hana Sychrová

**Affiliations:** 1 Department of Membrane Transport, Institute of Physiology of the Czech Academy of Sciences, 14220 Prague 4, Czech Republic; 2 Department of Microbiology and Parasitology, Faculty of Pharmacy, Complutense University of Madrid and IRYCIS, Madrid, Spain; 3 Department of Molecular Biosciences, The Wenner-Gren Institute, Stockholm University, SE-10691 Stockholm, Sweden; 4 Department of Microbiology, University of Szeged Interdisciplinary Excellence Centre, Szeged, Hungary; 5 MTA-SZTE “Lendület” “Mycobiome” Research Group, University of Szeged, Szeged, Hungary

**Keywords:** Pathogens, Cell growth, Fungal infection

## Abstract

The absence of high-affinity potassium uptake in *Candida glabrata*, the consequence of the deletion of the *TRK1* gene encoding the sole potassium-specific transporter, has a pleiotropic effect. Here, we show that in addition to changes in basic physiological parameters (e.g., membrane potential and intracellular pH) and decreased tolerance to various cell stresses, the loss of high affinity potassium uptake also alters cell-surface properties, such as an increased hydrophobicity and adherence capacity. The loss of an efficient potassium uptake system results in diminished virulence as assessed by two insect host models, *Drosophila melanogaster* and *Galleria mellonella*, and experiments with macrophages. Macrophages kill *trk1*Δ cells more effectively than wild type cells. Consistently, macrophages accrue less damage when co-cultured with *trk1*Δ mutant cells compared to wild-type cells. We further show that low levels of potassium in the environment increase the adherence of *C*. *glabrata* cells to polystyrene and the propensity of *C*. *glabrata* cells to form biofilms.

## Introduction

The incidence of fungal infections of the *Candida* genus has increased in recent decades, and among them *Candida glabrata* is classified as the second most commonly isolated yeast in the majority of patient populations studied^1–4^. The reasons for this upswing are on the one side medical progress by itself, which has increased life expectancy, but also the niches where opportunistic fungi can develop. The use of antibiotics, catheters or transplantation therapies has generated a susceptible population that has helped fungal pathogens come to the front line of clinical problems in developed countries^5,6^. The success of various yeast species as pathogens depends on their ability to adapt to the environmental stresses they encounter within the diverse niches they occupy in the human host^7^. For many years, pathogenic yeasts were assumed to passively contribute to the establishment of infection, but nowadays, it is well known that these organisms dynamically participate in the disease process through mechanisms of aggression, called virulence factors. Among these factors, the ability to evade host defenses, adherence, biofilm formation and the production of tissue-damaging hydrolytic enzymes play a crucial role^8,9^.

*Candida glabrata*, a member of the WGD (Whole Genome Duplication) yeast family and a close relative of *Saccharomyces cerevisiae*, is an opportunistic yeast pathogen that is distantly related to the CTG clade of yeast (yeast species translating the CUG codon as serine instead of leucine), which includes most of the pathogenic *Candida* species^10^. Its high stress resistance and high adhesion capacity are characteristics that make *C*. *glabrata* a serious pathogen for humans^11^. The ability of *C*. *glabrata* to respond to changes in environmental conditions with rapid transcriptional reprogramming, together with its robust resistance to both nutrient starvation and oxidative stress^12^, are properties that provide *C*. *glabrata* a competitive advantage when nutrient availability is low, such as on mucosal surfaces or within phagosomes after engulfment by phagocytic cells. In the latter case, *Candida* cells are also exposed to reactive oxygen species and reactive nitrogen species^13^, moreover, host immune cells also activate intracellular ion currents that might expose *Candida* cells to cationic and osmotic stresses^14^. Given this situation, it is not surprising that *C*. *glabrata*, as well as other *Candida spp*., have evolved a robust tolerance to cationic/osmotic, oxidative and nitrosative stresses^15,16^.

Adherence is one of the crucial steps in the establishment of fungal infections, and it is a feature that enables *C*. *glabrata* to adhere to host epithelial tissues and other surfaces, e.g., catheters. This trait is coupled to virulence, and is mediated by cell-wall associated proteins termed adhesins, which belong to diverse protein families. Several studies have demonstrated that *C*. *glabrata* has a large repertoire of adhesins^17,18^, which facilitate its ability to colonize humans. Another factor, considered important for virulence, and related to adherence, is cell-surface hydrophobicity (CSH), which depends on the cell-wall composition and architecture. The relative CSH of *C*. *glabrata* is thought to be more extensive than that of *Candida albicans*, the best studied yeast pathogen^19^. After the adhesion to host tissues or other surfaces within the host, yeast cells grow and develop a biofilm, i.e., a population of cells embedded within a self-synthesized extracellular matrix^20^. *C*. *glabrata* biofilms, composed of a compact monolayer or multilayer of only blastospores^21^, are extremely resistant to antifungal therapies, being able to withstand much higher concentrations of antifungal drugs than planktonic cells, and thus making *C*. *glabrata* biofilm infections extremely challenging to treat^22^.

In addition to virulence factors, such as adhesion and biofilm formation, fitness traits such as rapid adaptation to fluctuations in environmental pH, metabolic flexibility, powerful nutrient acquisition systems and robust stress response machineries, influence fungal pathogenicity and support the ability of *Candida spp*. to infect diverse host niches. Many studies have shown that the disruption of various signaling and/or metabolic pathways has led to a diminished virulence and pathogenicity of yeast cells^23^. Also important in the establishment of the infection, is the expression of efficient and robust systems for the uptake of different compounds serving as carbon or nitrogen sources, or providing necessary metal ions, such as iron or zinc^24–27^.

Potassium is the most abundant metal cation in all organisms. Due to its low toxicity and high ability to bind water, it has many general physiological functions in all cells. Consistent with its importance, cells spend a lot of energy to accumulate potassium in relatively high concentrations. In general, it is indispensable for establishing intracellular turgor, which is necessary for cell growth and expansion, and for the compensation of negative charges of many macromolecules, including DNA, RNA and polyphosphates. In yeast, potassium fluxes and accumulation are also indispensable for the regulation of intracellular pH and membrane potential, for the activation of various enzymes, for protein synthesis and many other functions^28–30^. Yeast cells have three types of potassium uptake systems, which allow cells to concentrate potassium to a 200–300 mM concentration from the environment with as low as micromolar concentrations of potassium salts^28–31^. All three types of transporters exist in *Candida* species^32^. They share the same basic function (the uptake of potassium) but are very different from the mechanistic, structural and phylogenetic points of view. Moreover, none of them has a homologue in mammalian cells. *C*. *albicans* has all three types of these transporters, i.e., the Trk uniporter, Hak potassium-proton symporter and Acu potassium-influx ATPase^33^. This might be an advantage in proliferating in host niches with relatively low potassium concentrations, or in formation and rapid growth of hyphae, a process which needs a high intracellular turgor^34^.

The *C*. *glabrata* genome has only a single potassium-uptake system encoded by *TRK1*. This is surprising, since most yeast species have at least two types of potassium uptake systems (usually Trk and Hak, reviewed in^29^) and the closely related *S*. *cerevisiae* has two *TRK* genes^35,36^. The existence of only one potassium-uptake system in *C*. *glabrata* and the need of yeast cells to accumulate high intracellular K^+^ concentrations to ensure cell growth and division, turned our attention to the characterization of *C*. *glabrata* Trk1 and the phenotypes of its absence^37^. We showed that *TRK1* indeed encodes an efficient potassium uptake system in *C*. *glabrata* cells. The expression of *CkTRK1* is low and constitutive, similarly as the expression of *TRK1* in *S*. *cerevisiae*^28^. The deletion of *TRK1* has a pleiotropic effect on the cell physiology, not only affecting the ability of *trk1Δ* mutants to grow at low potassium concentrations, but also their tolerance to toxic alkali-metal cations and cationic drugs, as well as the ability to maintain their membrane potential and intracellular pH. Taken together, our current understanding is that the sole potassium uptake system of *C*. *glabrata* is critical to its physiology and fitness, and suggests that potassium uptake may affect virulence. In this report we compare *C*. *glabrata* strains lacking *TRK1 (trk1*Δ) with its wild-type parent, focusing on traits related to virulence, such as cell surface properties, the ability to cause infections in two insect host models and challenge to phagocytosis by macrophages.

## Results

### Lack of Trk1 increases cell surface hydrophobicity and adherence capacity

**Figure 1:**
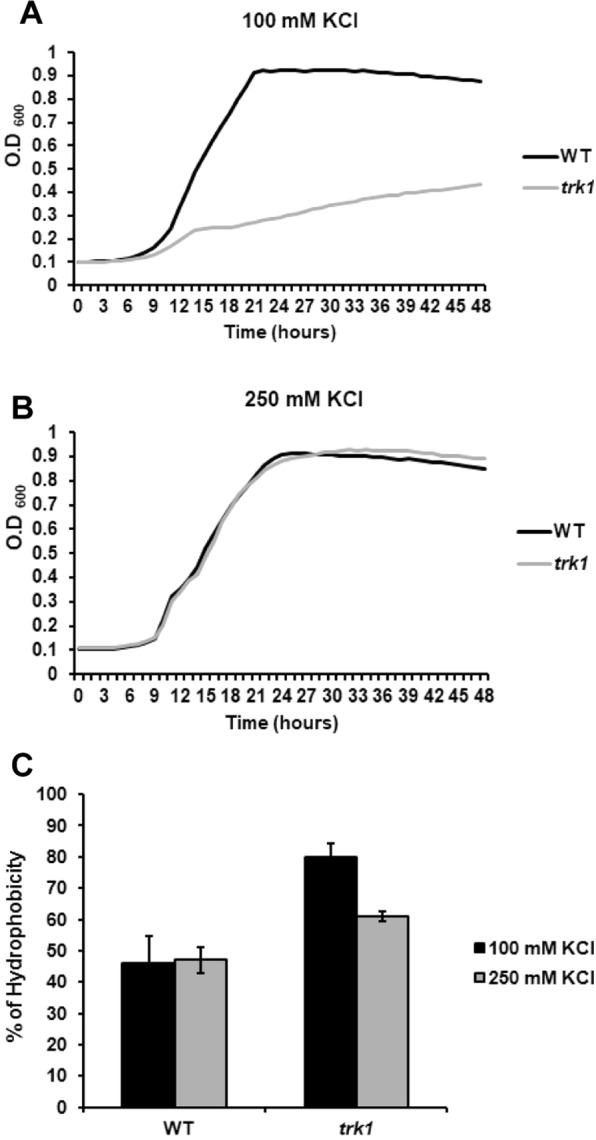
Hydrophobicity of *C*. *glabrata* cell surface depends on Trk1 function. The growth of cells was monitored in YNB-F medium supplemented with 100 mM (**A**) or 250 mM (**B**) KCl. The relative hydrophobicity (**C**) of the wild-type and *trk1Δ* cells grown in YNB-F media supplemented with 100 or 250 mM KCl was estimated as described in Methods.

The yeast cell wall has a highly dynamic structure, and its composition is tightly controlled not only during the cell cycle, but also in the different growth phases and during the adaptation to environmental changes. Alterations in cell-wall composition have consequences such as the modification of CSH or altered susceptibilities to cell-wall targeted drugs^17^. To elucidate whether the observed changes in membrane potential and susceptibility to cationic drugs of the *trk1*Δ mutant^37^ are also reflected in the cell surface properties, we compared the CSH of wild-type and *trk1Δ* cells. The relative CSHs were estimated with cells grown under two conditions – in the presence of 100 mM KCl (a concentration at which *trk1*Δ cells can grow but clearly experience low-potassium stress, Fig. 1A) and 250 mM KCl (a concentration at which both the wild type and *trk1*Δ strains grow similarly, Fig. 1B). As shown in Fig. 1C, the mutant strain exhibited a higher hydrophobicity at both of the tested KCl concentrations. As expected, the CSH of the mutant was higher than that of the wild type when cells were grown with 100 mM KCl, but strikingly, a significantly higher CSH was observed for the *trk1*Δ cells in 250 mM KCl, i.e., a concentration close to the physiological intracellular concentration (approx. 280 mM in exponentially growing *C*. *glabrata* cells^38^). This latter observation suggested that the absence of Trk1-mediated transport leads to permanent changes in the cell wall, and consequently, to changes in cell surface hydrophobicity. Changes in cell-wall or plasma-membrane composition may be reflected in increased cell sensitivity to compounds such as Congo red or SDS. For this reason, we tested whether the *trk1*Δ mutant exhibited alterations in growth when these two compounds were added to the media. No significant differences were observed between the growth of the wild-type strain and the mutant on solid media supplemented with 250 mM KCl and SDS (up to 0.05%) or Congo red (up to 400 µg/ml) (data non-shown).

**Figure 2:**
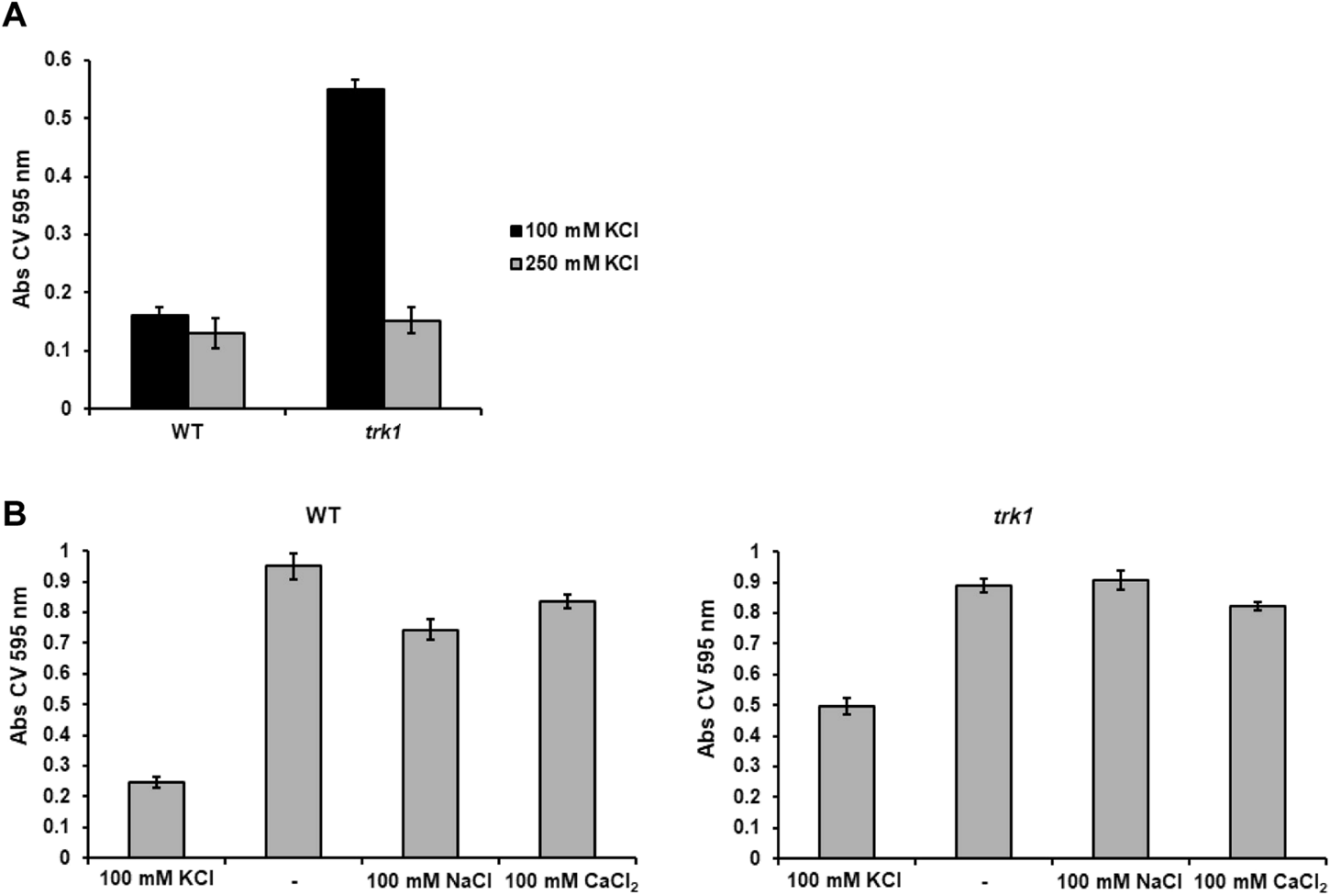
*C*. *glabrata* adherence capacity depends on amount of potassium in environment. (**A**) The adherence of the wild-type and *trk1Δ* cells grown in YNB-F media supplemented with 100 or 250 mM KCl to polystyrene was estimated as described in Methods. (**B**) Cells pregrown in YNB-F media supplemented with 100 mM KCl were transferred to YNB-F media without extra added KCl (−) or supplemented with NaCl or CaCl_2_ as indicated, and their adherence capacity was estimated as described in Methods.

Alterations in potassium fluxes and homeostasis may affect the adhesion capacity of *C*. *glabrata* to polystyrene. We therefore compared the adhesion properties of the wild-type and the *trk1*Δ strains grown in the presence of 100 or 250 mM KCl. As shown in Fig. 2A, the adhesion capacity of the wild type was almost the same under both growth conditions. On the other hand, the adhesion capacity of the mutant changed with the availability of potassium in the growth medium. As compared to wild-type cells, the *trk1*Δ cells adhered similarly at 250 mM KCl, but when grown in 100 mM KCl, their adhesion capacity was significantly increased. Thus, the higher CSH of the *trk1*Δ mutant (Fig. 1C) was not accompanied by an increased adhesion when the cells were grown at 250 mM KCl. To verify the differences, the same experiments were performed with another independently constructed *trk1*Δ mutant and the same results were obtained (data shown only for one of the two mutant strains).

These results led us to test the specific role of potassium in cell adherence. Cells were grown in 100 mM KCl (non-stress conditions for the wild-type strain, low-potassium stress for the *trk1*Δ mutant), and then shifted to YNB-F without the addition of any salt (YNB-F contains 15 µM K^+^) or supplemented with either 100 mM KCl, 100 mM NaCl, or 100 mM CaCl_2_. The results (Fig. 2B) show that when cells were shifted from YNB-F with 100 mM KCl to YNB-F lacking salt (−), both wild-type and *trk1Δ* cells increased their adhesion to polystyrene significantly, and to a similar level. This increase was also present for both strains when cells were transferred to media with 100 mM NaCl or CaCl_2_. The results suggest that *C*. *glabrata* adherence is a potassium-specific phenotype.

### Potassium influences formation of biofilms

**Figure 3:**
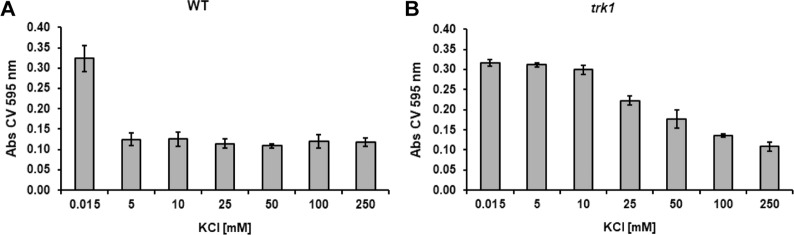
Limited external potassium increases biofilm formation. Wild-type and *trk1Δ* cells were pregrown in YNB-F supplemented with 250 mM KCl, then the adherence step in the same medium occurred for 2 h, non-adhered cells were washed out, fresh YNB-F medium supplemented with KCl as indicated was added, and the biofilm formation was estimated after 48 h as described in Methods.

It is believed that the formation of mature biofilms and the production of extracellular matrix is strongly dependent on species, strain, and environmental conditions such as pH, medium composition and oxygen availability^39^. As we observed a potassium-dependent increase in the adherence ability of *C*. *glabrata* cells, we speculated that the formation of a biofilm might also be dependent on the availability of potassium cations. Cells were pre-grown in YNB-F supplemented with 250 mM KCl, i.e. under conditions in which both strains exhibited similar adherence (Fig. 2A), transferred to polystyrene plates, and after a two-hour incubation, the medium was replaced with a series of media containing KCl at concentrations ranging from 15 µM to 250 mM. For the wild-type strain, a significant biofilm was formed only when grown in YNB-F without added potassium (0.015 mM; Fig. 3A); similar low levels of biofilm formation were observed at KCl concentrations between 5–250 mM. By contrast, the *trk1*Δ mutant formed biofilms over a wide range of KCl concentrations; significant biofilms formed even at 100 mM KCl (Fig. 3B). At the higher KCl concentrations tested, the *trk1*Δ mutant produced significantly larger biofilms than the wild type. These results suggested that potassium availability and its transport to cells have a direct effect on biofilm formation. The capacity of *C*. *glabrata* to form biofilms was inversely proportional to the K^+^ concentration, suggesting that cellular stress resulting from potassium limitation promotes biofilm formation.

### Absence of Trk1 makes *C*. *glabrata* less virulent in insect models

The higher cell-surface hydrophobicity, as well as the higher adherence and biofilm formation of *trk1*Δ cells in the presence of a standard potassium concentration suggested that the mutant cells might be more virulent than the wild-type. On the other hand, the inability of the *trk1*Δ mutant to proliferate in media with a potassium concentration close to the concentrations in the host (a few mM extracellularly) would correspond to a lower virulence of mutants lacking an active potassium uptake system. To elucidate the relationship between the absence of Trk1 and virulence, we performed a series of experiments using two insect models, *Drosophila melanogaster* and *Galleria mellonella*. These invertebrate models are capable of reproducing clinical features seen in human infections with remarkable fidelity^40–42^.

**Figure 4:**
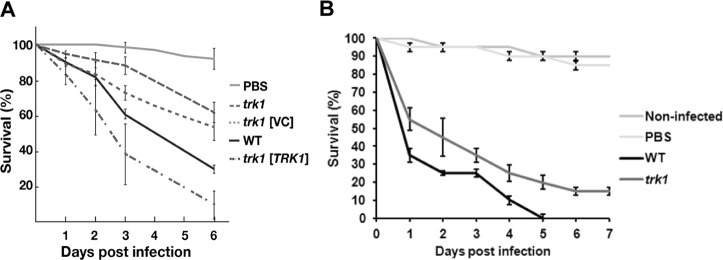
*C*. *glabrata* cells lacking Trk1 are less virulent in insect models. *D*. *melanogaster* Bom^Δ55C^ flies (**A**) were infected with *C*. *glabrata* wild-type or *trk1* mutant strains without a plasmid vector (*trk1*), with an empty vector control (*trk1* [VC]), or with a vector expressing *TRK1* (*trk1* [*TRK1*]). Survival was monitored for the indicated period of days. Survival curves were obtained from the Cox proportional hazards model with data from 4 biological replicates (N = 4; bars represent standard error). (**B**) Survival of *G*. *mellonella* larvae infected with *C*. *glabrata* wild-type or *trk1* mutant strains was monitored for the indicated period of days. Data from 3 biological replicates (N = 3; bars represent standard error). PBS was used to control wounding.

When introduced into *D*. *melanogaster*, fungal cells activate the Toll signaling pathway, triggering a robust induction of innate immune effectors, including the family of twelve *Bomanin* genes. The Bomanins, small secreted peptides, bestow resistance to multiple microbial pathogens, including *C*. *glabrata*. In contrast to wild-type flies, *Bom*^*Δ55C*^ flies lacking10 Bomanins exhibit decreased survival upon *C*. *glabrata* infection^43^, similar to mutational inactivation of the essential Toll pathway in MyD88 flies^44^. Figure 4A shows the killing curves obtained with Bom^Δ55C^ flies infected with wild-type and *trk1*Δ *C*. *glabrata* strains. It is clearly evident that flies infected with the mutant strain survived better than those infected with the wild type. The introduction of plasmid encoded *TRK1*, but not the empty plasmid (VC; vector control), into the *trk1*Δ mutant restored full virulence. When the MyD88 flies were used, similar results were observed, i.e. lower virulence of the *trk1Δ* mutant (data not shown). For the assays with *G*. *mellonella*^45^, two temperatures were used: 30 and 37 °C. We obtained similar results in both cases, and those obtained from assays at 37 °C are shown in Fig. 4B. Although both *Candida* strains killed the larvae quite efficiently, it was evident that the infection with *trk1*Δ cells was less severe than that with the wild-type *C*. *glabrata* cells.

Together, the virulence assays in the insect hosts confirm that the loss of Trk1-mediated high-affinity potassium uptake results in attenuated virulence of *C*. *glabrata*. Clearly, the *trk1Δ* mutant failed to accumulate sufficient potassium for cell growth and division within the host.

### Absence of Trk1 results in increased clearance of *C*. *glabrata* cells by macrophages

The reduced virulence of *trk1*Δ mutant cells prompted us to examine the host-pathogen interaction with THP-1 macrophages. Macrophages are immune cells important in the recognition and destruction of pathogens^46,47^. These primary immune cells contain, as all mammalian cells, a high concentration of potassium (>100 mM). However, the levels of potassium in phagosomes have not been determined. Initially, the internal phagosome microenvironment should mirror the low extracellular concentration until it fuses with lysosomes, at which point the potassium levels should increase. Thus, there are two possibilities. The initial low levels of potassium may compromise growth of *trk1*Δ cells, which may affect survival. Alternatively, lysosomal fusion is rapid and *trk1*Δ cells experience sufficiently high levels of potassium to enable growth at rates comparable to wild-type.

**Figure 5:**
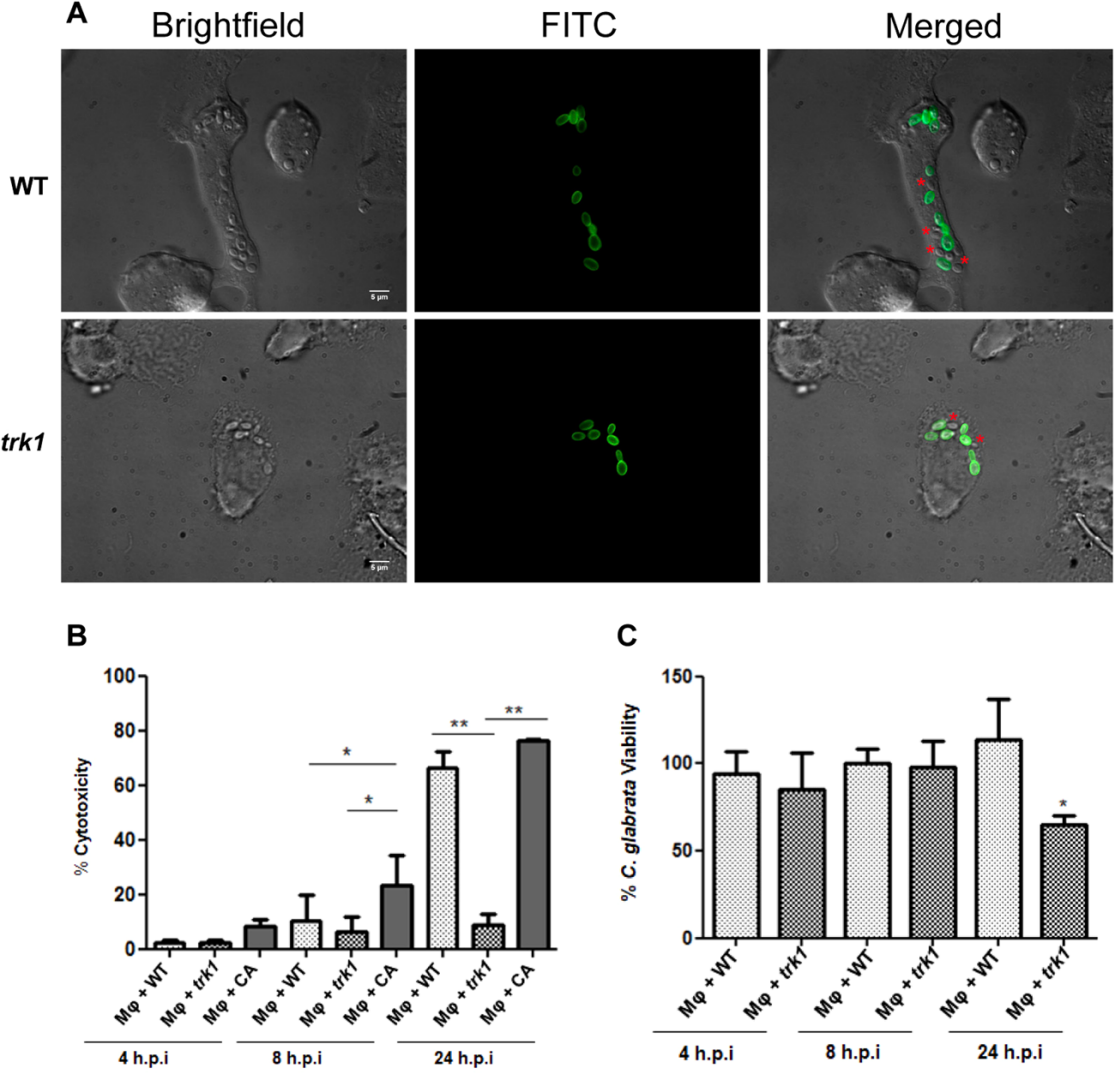
*C*. *glabrata* wild-type and *trk1Δ* cells interaction with human THP1 macrophages. (**A**) THP1 macrophages were incubated with FITC stained (green) *C*. *glabrata* cells, and fluorescence microcopy was used to evaluate yeast cell interaction with macrophages within 6 hours of incubation. The dye is not transferred to the daughter cells, allowing to differentiate mother cells (green) and daughter cells (red asterisks) in the merged channel. (**B**) Cytotoxicity mediated by *C*. *glabrata* wild-type (WT) and *trk1Δ* cells in THP1 macrophages was evaluated by LDH measurement. *C*. *albicans* wild-type strain SC5314 (CA) was used as a positive control. (**C**) Candidacidal activity of THP1 macrophages against *C*. *glabrata* wild type (WT) and *trk1Δ* was evaluated and expressed as a percentage of yeast viability. A lower viability of the yeast cells represents a higher candidacidal activity of the macrophages.

Our initial observations regarding the growth and division of *C*. *glabrata* cells interacting with macrophages indicated that the *trk1*Δ strain exhibited slower growth in comparison to wild type (Fig. 5A). The growth of one hundred *C*. *glabrata* cells interacting with macrophages was monitored during 6 hours. Thirty percent of wild-type cells had duplicated, whereas only 20% of *trk1*Δ cells had duplicated. The lower level of growth of *trk1*Δ mutant cells suggested that the interaction between the macrophages and both *C*. *glabrata* strains differ and that a high affinity potassium uptake is required for proper yeast proliferation in this condition. These findings prompted us to perform further experiments.

To assess fungal cell-induced damage to macrophages during co-culturing we measured LDH release (% cytotoxity, Fig. 5B). *C*. *albicans*, known to cause high levels of damage, was used as a control^48^. The *trk1*Δ mutant caused almost no damage to macrophages as compared to wild type (the difference was already observable after 8 h, and clearly obvious after 24 h, Fig. 5B). Both *C*. *glabrata* strains caused less damage than *C*. *albicans*, particularly at the earlier timepoint, which correlates with the different strategies that these fungi employ after being engulfed by macrophages. *C*. *albicans* rapidly forms hyphae, damages macrophages and escapes, while *C*. *glabrata* seems to be adapted to longer stays inside the macrophages^47^.

These results suggested that the candidacidal activity of the macrophages towards the *trk1*Δ strain may be higher. This was confirmed in the experiments shown in Fig. 5C. The macrophages killed *trk1*Δ cells more effectively than wild type, which was statistically significant after 24 h. These finding indicates that the loss of high-affinity potassium uptake impairs growth of *C*. *glabrata* inside macrophages and makes fungal cells more sensitive to killing by macrophages.

### Cytokine production upon interaction of macrophages with *C*. *glabrata* cells

Cytokine production and secretion by macrophages is important in mediating the response of the immune system and to the outcome of microbial infections^49^. We assessed the cytokine profiles of macrophages after co-culture with the *C*. *glabrata* strains. We measured the release of 3 pro-inflammatory (IL-12, TNF-α and IL-1 β) and 1 anti-inflammatory (IL-10) cytokines. We were unable to detect secretion of IL-12 nor IL-10 cytokines in our experiments (data not shown). TNF-α was secreted, but there was no difference in secretion levels between control macrophages and macrophages with *C*. *glabrata* wild-type or *trk1*Δ cells (data not shown). The secretion of pro-inflammatory cytokine IL-1β was elevated similarly in the presence of either wild-type or *trk1*Δ (Supplementary Fig. 1). These largely negative results are in agreement with previous reports regarding *C*. *glabrata* macrophage interactions^50^, with the cytokine patterns in murine infections^51^ and with the previously described low level induction of MAP kinase phosphorylation^52^.

## Discussion

In our previous work we showed how the deletion of *TRK1*, encoding the sole potassium transporter in *C*. *glabrata*, has a pleiotropic effect on cell physiology, affecting the membrane potential, the intracellular pH and the tolerance to cationic drugs^37^. Based on this knowledge, we hypothesized that the altered physiological parameters of the mutant would also effect *C*. *glabrat*a virulence. To test this notion we performed a series of experiments to document whether a *C*. *glabrata* strain lacking Trk1 exhibits altered virulence characteristics and a reduced capacity to induce virulent infections in insect hosts models and to kill and evade macrophages.

Initially, we focused on two closely related aspects known to be important determinants in virulence, cell-surface hydrophobicity (CSH) and adhesion capacity^17,18^. As shown in Fig. 1C, we observed a significant difference in CSH when comparing the wild-type strain and *trk1*Δ mutant. The wild type exhibited no differences in CSH at the two tested potassium concentrations (100 and 250 mM), suggesting that the composition of the cell wall and physico-chemical properties of the cell surface do not change in this KCl concentration range. It is worth noting that wild-type grows well at both much lower and higher extracellular potassium concentrations^37,38^. In comparison to the wild-type, the *trk1*Δ strain exhibited a significant increase in its CSH (Fig. 1C). Moreover, there was a clear increase in the CSH of the mutant at 100 mM KCl compared to its CSH at 250 mM KCl in the growth media. The obtained results suggest that the deletion of *TRK1* causes a basal stress that has persistent consequences on the composition of the cell wall, thus influencing the hydrophobicity of mutant cells even under conditions supporting an apparently normal growth rate (Fig. 1B). A similar situation was found when measuring the membrane potential of *C*. *glabrata* wild-type and *trk1*Δ cells grown in the presence of 250 mM KCl. Mutant cells were always relatively hyperpolarized, indicating that the deletion of *TRK1* affects the physiology parameters permanently and not only under low-potassium stress^37^.

As with the CSH, the adherence of the wild-type cells did not seem to be significantly influenced at the two KCl concentrations used (100 and 250 mM; Fig. 2A). In striking contrast, although the adherence capacity of the *trk1*Δ mutant grown in the presence of 250 mM KCl was very similar to that of the wild type, the *trk1*Δ exhibited a dramatic increase in its adhesion capacity when a suboptimal 100 mM concentration of KCl was used (Fig. 2A). To elucidate whether the observed phenotypes of higher adhesion were potassium-specific or related to a general concentration of cations in the growth media, we tested adhesion in low K^+^ media supplemented with Na^+^ and Ca^+^. Surprisingly, we observed a significant increase in the cell adhesion to polystyrene plates for both wild type and *trk1*Δ strains (Fig. 2B). This result means that the low concentration of potassium, and not the presence of other cations, is responsible for the observed increase in adhesion capacity. Consistent with this conclusion, the *trk1*Δ strain exhibited significantly more adhesion than wild type in media containing 100 mM KCl. As far as we know, this is the first time that the dependence of *C*. *glabrata* adhesion capacity on the amount of potassium cations in the external medium has been demonstrated.

The observed increase in the adherence of the wild-type strain in YNB-F without extra added KCl made us hypothesize that this phenomenon might have consequences in biofilm formation, enabling the cells to cope better with the limited amount of available potassium, as the extracellular matrix may trap and concentrate the potassium. To test this hypothesis, an experiment was performed at different concentrations of potassium (Fig. 3). At low KCl (15 µM) concentrations the biofilm formation of wild type correlated with the higher adherence, which is not surprising, since adhesion is the first phase of biofilm formation^20^. As shown in Fig. 3, the biofilm biomass of the wild-type strain was greatly reduced when potassium levels increased. By contrast, the *trk1*Δ strain persisted in establishing biofilms even up to 100 mM KCl; the progressive decrease in the formation of a biofilm in parallel with an increase in potassium concentration reinforces the idea that the change from planktonic cells to cells that form biofilms depends on the intracellular potassium supply. The fact that biofilm formation is strongly dependent on potassium, suggests that biofilm formation is a stress response and that growth in biofilms enables cells to maintain a critical concentrations of this essential alkali metal cation.

As was shown in our previous work^37^, the *trk1*Δ mutant exhibits several physiological parameters that reduce its growth and fitness, which likely affect its virulence properties. For this reason, we examined whether the phenotypes resulting from *TRK1* deletion would also have an effect on the virulence of the mutant strain. The results obtained with experiments carried out in two model host systems, *D*. *melanogaster* and *G*. *mellonella* (Fig. 4), provided support for this notion; the *trk1*Δ strain exhibited significantly attenuated virulence compared to the wild type in *Bom*^*Δ55C*^ flies. Although *D*. *melanogaster* is considered to be the most suitable insect model alternative to murine infection models^42^, the use of *G*. *mellonella* gave similar results.

In summary, our data show that the deletion of *TRK1* has a pleiotropic effect on the physiology of *C*. *glabrata*, which affects its ability to colonize infected hosts. The reason for impaired virulence of the mutant strain lacking a high-affinity potassium-specific transporter is likely due to the inability to take up and maintain physiological intracellular levels of potassium, an essential cation. The resulting stress and impaired growth of the *trk1*Δ mutant enables the host immune system to be more effective at overcoming the infection and clearing the fungal cells from the host. Consistently, the experiments carried out with macrophages demonstrated that in comparison to wild type, *trk1*Δ strain grew less efficiently within phagosomes (Fig. 5A), inflicted less damage to the macrophages (Fig. 5B), and was more readily killed (Fig. 5C). Macrophages are known to actively sequester micronutrients from invading microorganisms^53^, and the lack of Trk1 as the sole high-affinity and specific potassium uptake system may represent an additional handicap that promotes the higher susceptibility of the mutant to being killed by the macrophage. Additionally, we previously demonstrated that the *trk1Δ* mutant is sensitive to low external pH^37^, which is another aspect that may contribute to the observed increased fungicidal capacity of the macrophages against the mutant, since one of the strategies of the defense cells for fighting invading microorganisms is the acidification of their phagosomes^52^. A high-affinity potassium uptake seems to be crucial for *C*. *glabrata* physiology and virulence thus highlighting the unique K^+^ transporter in *C*. *glabrata* cells as a potential target for the development of a new antifungal drug.

## Methods

### Yeast strains and growth media

The *C*. *glabrata* reference strain ATCC 2001 and its derivative lacking the *TRK1* gene^37^ were used in this study. *C*. *albicans* SC5314 was used for LDH measurements. Yeast cells were propagated in YPD (1% yeast extract, 2% peptone, 2% glucose, 2% agar for solid media) or YNB-F (0.17% YNB without amino acids, ammonium sulfate and potassium (ForMedium) supplemented with 0.4% ammonium sulfate, 2% glucose and adjusted to pH 5.8 with NH_4_OH; potassium concentration approx. 15 μM) media at 30 °C. When necessary, the media were supplemented with indicated amount of KCl.

### Cell surface hydrophobicity (CSH)

CSH was determined as the relative distribution of yeast cells in a two-phase system consisting of an aqueous phase and the organic solvent hexadecane^17,54^. When cultures in YNB-F supplemented with 250 or 100 mM KCl reached OD_600_ = 1.5, cells were harvested, washed twice with distilled water, adjusted to OD_600_ = 1. Aliquots (1.5 ml) were transferred to a glass tube with (A_1_) and without (A_0_) 100 µl of hexadecane. Glass tubes were then mixed by gentle vortexing for 30 seconds. The two phases were allowed to separate for 2 minutes at room temperature, 1 ml of the aqueous phase of each tube was carefully transferred to a cuvette and the OD_600_ was measured. The percentage of hydrophobicity was calculated as Hydrophobicity (%) = [1 − (A1/A0)] × 100.

### *In vitro* adhesion capacity

Overnight cultures grown in YNB-F supplemented with 250 or 100 mM KCl (OD_600_ ≈ 1.5) were adjusted to OD_600_ = 1 with fresh growth medium. Three wells of a flat-bottom polystyrene 96-well microtiter plate were filled with 200 µl of each cell suspension (adapted from^18^). Growth media without cells were used as negative controls. Adhesion was allowed to occur at 30 °C for 2 hours. After removing the medium, non-adherent cells were removed by washing three times with sterile water. Adhered cells were fixed with 200 μl of methanol, and plates were incubated at room temperature for 10 minutes. After washing three times with water to eliminate methanol, cells were stained with crystal violet (CV). 200 µl of 1% CV solution were added to each well, and plates were incubated at 37 °C for 20 minutes. After staining, the excess of CV was removed by washing three times with water. After adding 200 µl of 33% acetic acid to solubilize cell-bound CV, the staining intensity was measured as the OD_595_ using a 96-well plate reader (BioTek). The obtained values were normalized after subtracting the background level of CV staining without cells. For the potassium specificity assay, wild-type and *trk1*Δ cells were grown in YNB-F supplemented with 100 mM KCl till the cultures reached an OD_600_ of around 1. Cells were harvested, washed twice and resuspended to OD_600_ = 0.4 in YNB-F without KCl addition, or in YNB-F supplemented with 100 mM KCl, NaCl or CaCl_2_. After this step, the adhesion protocol described above was followed.

### Biofilm formation

For the study of biofilm formation, a modified version of the protocol of^55^ was used. Both strains were grown in YNB-F supplemented with 250 mM KCl at 30 °C overnight, then adjusted to OD_600_ = 0.4 with fresh medium. For adhesion, three wells of a polystyrene microtiter plate were filled with 200 µl of the cell suspension for each set of biofilm formation conditions. After two hours of incubation at 30 °C, the medium was removed, free cells were washed out twice with water, and YNB-F supplemented with various concentrations of KCl (15 µM–250 mM) was added to the adhered cells. Plates were further incubated at 37 °C for 48 hours, and the quantification of the biofilm formation was performed after the staining with CV described above. As a control after the adhesion phase, the number of adhered cells of wild-type and *trk1*Δ mutant grown in 250 mM KCl was estimated by CV staining as described above to ensure that the number of adhered cells was the same for both strains. No significant differences were observed in the initial number of adhered cells.

### Virulence in insect models

For testing the virulence of *C*. *glabrata* strains, *Drosophila melanogaster* flies and *Galleria mellonella* larvae were used. *D*. *melanogaster* stocks were maintained on instant mashed potato agar medium at 25 °C. *Bom*^*Δ55C*^ flies, carry a 9 kb TALEN-induced deletion that removes a cluster of 10 *Bom* genes on chromosome 2^43^. The *MyD88* mutant strain lacks an adaptor protein functioning downstream of Tl receptor^44,56^.

The overnight yeast precultures were diluted in fresh YPD to OD_600_ = 0.15 and incubated at 30 °C until the OD_600_ reached 1.0. Aliquots of the cultures (1 ml) were harvested, washed once with phosphate-buffered saline (PBS; pH 7) and resuspended in 1 ml of PBS. Male and female flies, 1–5 days old, were injected with approximately 50 nl of fungal cell suspensions (approx. 500 cells/fly) using a fine glass capillary needle with a micro-injector (TriTech Research, USA). Cohorts of 30 flies were injected and maintained in separate vials. Four biological replicas (independently prepared fungal preparations derived from individual colonies) and 12 technical replicates per strain were conducted. Infected flies were maintained at 29 °C for up to six days after infection and the number of surviving flies was noted on a daily basis.

*G*. *mellonella* larvae were from Mous Live Bait, The Netherlands. One day before the infection experiment, ten groups of ten *G*. *mellonella* larvae were placed in ten Petri dishes containing sawdust and incubated at 30 or 37 °C to acclimatize them to the conditions of the experiment. *C*. *glabrata* cells were grown in 15 ml of YPD supplemented with 100 mM KCl overnight. The day of infection, 10 ml of the *C*. *glabrata* cultures were harvested, washed twice with PBS and resuspended in 2 ml of PBS. After measuring OD_600_, cell suspensions of both strains containing approx. 5 × 10^7^ cells/100 µl were prepared, and 10 µl, i.e. 5 × 10^6^ cells, were injected into one of the last pro-legs of the larvae with an insulin 29 G U-100 needle. Twenty individuals per yeast strain were infected. Three biological replicas were conducted. Untouched larvae and larvae injected with 10 µl PBS were used as controls. Larvae were kept in Petri dishes with sawdust, and live larvae were scored daily for 7 days. Larvae were considered dead when not responding to touch.

### Interaction with macrophages

To quantify yeast replication upon interaction with macrophages, *C*. *glabrata* strains were grown in YNB430 F supplemented with 250 mM KCl and labelled with 100 µg/ml FITC (Sigma-Aldrich) in carbonate buffer (0.1 M Na_2_CO_3_, pH 9.0) for 30 min at 37 °C, followed by washing with PBS. TPH-1 macrophages were allowed to adhere to coverslips within a 24-well plate, infected at a MOI (multiplicity of infection; macrophage:yeast) 2:1 with labelled yeast strains for 30 min, washed to remove unbound yeast cells, and incubated at 37 °C and 5% CO_2_ for 6 hours. Cells were fixed with 4% paraformaldehyde at 37 °C for 10 min. As FITC is not transferred to daughter cells, it was possible to differentiate mother and daughter cells. Replication was quantified by fluorescence microscopy scoring FITC-stained and not-stained cells for at least 100 yeast cells.

### THP-1 cell culture and macrophage differentiation

The human acute monocytic leukemia cell line (THP-1) was cultured in DMEM medium supplemented with antibiotics (penicillin 10000 U/ml-streptomycin 10000 U/ml and 10% heat-inactivated fetal bovine serum (FBS) at 37 °C in a humidified atmosphere containing 5% CO_2_. THP1 cells were centrifuged and resuspended in fresh DMEM. Phorbol 12-myristate 13-acetate (PMA) was added at a final concentration of 0.03 µg/ml and cells were counted. Then, 1 × 10^5^ THP-1 cells were seeded onto 24-well plastic plates and 5 × 10^4^ cells were seeded onto a 96-well plate and left to differentiate for 48 hours. The medium was replaced with fresh medium on the day of the interaction. DMEM without phenol red supplemented with 1% of FBS was used for the LDH measurements,. For the assays of candidacidal activity and cytokine production, DMEM with phenol red and 10% FBS was used.

### *C*. *glabrata-*macrophage co-culture

For the interaction studies, THP-1 macrophages were incubated with *C*. *glabrata* cells at a MOI 1:1 and for the durations: 4 h, 8 h and 24 h.

### Macrophage damage assay

A colorimetric assay based on the measurement of LDH activity released by damaged cells was used (Roche). Experiments were performed in 96-well plate and the manufacturer’s instructions were followed. Briefly, Lysis buffer was added to the positive control cells 15 minutes before the end of the incubation time. To determine LDH activity, 100 µl of reaction mixture, (catalyst and dye solution) was added to each well on the 96-well plate and incubated for up to 30 min at room temperature and protected from the light. After this, 50 µl of stop solution was added to each well and absorbance measured at 490 nm. Cytotoxicity was calculated as follows:

$$Cytotoxicity\,( \% )=\frac{experimental\,value-low\,control}{high\,control-low\,control}\times 100$$

Three biological replicates were performed.

### Candidacidal activity

The candidacidal activity of the macrophages was estimated by colony-forming units (CFUs) counting by comparing both *C*. *glabrata* strains with and without interaction with macrophages. 24-well plates were used for this assay. Briefly, the DMEM of each condition was collected, sterile H_2_0 was added to each well and a syringe plunger was used to destroy the macrophages and to resuspend *C*. *glabrata* cells in each well. From the total volume collected, dilutions were made and wild-type cells were plated on YPD agar and *trk1Δ* cells on YPD agar supplemented with 100 mM KCl. Candidacidal activity was calculated by comparing the CFUs counted for *Candida* cells growing without the presence of the macrophages and the CFUs counted the interacting cells. Four biological replicates were performed.

### Determination of cytokine production

For cytokines measurements, macrophages from the THP1 cell line were incubated for 4, 8 and 24 hours in 24-well plates. Briefly, supernatants from THP-1 macrophages (untreated, LPS (1000 ng/ml), or *Candida*-treated) were collected. Afterwards, they were tested for cytokine production by ELISA using matched paired antibodies specific for IL-12p40, TNF-α IL-10 and IL-1β (Immunotools), and according to the manufacturer’s instructions. Cytokine production was measured spectrophotometrically at 450 nm in a total of 3 biological replicates.

### Statistical analysis

Statistical analysis was performed by doing a paired t-test. *p-value < 0.05, **p-value < 0.01.

## Supplementary information

Supplementary Figure 1
