# Supplementary material for: Trk1-mediated potassium uptake contributes to cell-surface properties and virulence of Candida glabrata
Source: Sci Rep. 2019 May 17;9:7529. doi: 10.1038/s41598-019-43912-1 (PMC6525180; doi:10.1038/s41598-019-43912-1)

Trk1-mediated potassium uptake contributes to cell-surface properties and virulence of *Candida glabrata*

Vicent Llopis-Torregrosa, Catarina Vaz, Lucia Monteoliva, Kicki Ryman, Ylva Engstrom, Attila Gacser, Concha Gil, Per O. Ljungdahl, Hana Sychrová

Supplementary Figure 1. Pro-inflammatory cytokine IL-1β secretion by macrophages after interaction with *C. glabrata* wild-type (WT) and *trk1*strains. LPS (1000 ng/ml) was used as a positive control.

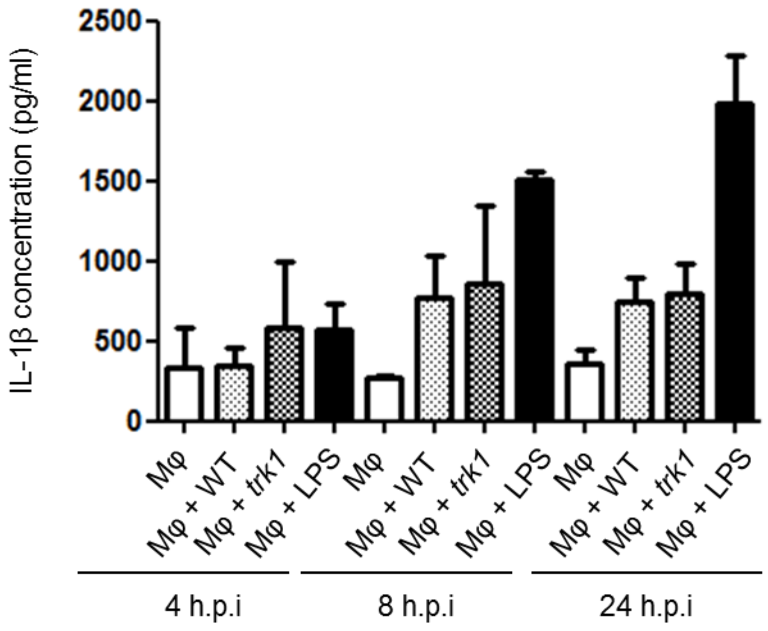

Supplement: Supplementary file 1 — Supplementary Figure 1 [file 41598_2019_43912_MOESM1_ESM.pdf]
